# Pertussis prevalence among adult patients with acute cough

**DOI:** 10.55730/1300-0144.5349

**Published:** 2022-01-11

**Authors:** Ahmet İLBAY, Mine Durusu TANRIÖVER, Pınar ZARAKOL, Ezgi Çalışkan GÜZELCE, Hatice BÖLEK, Serhat ÜNAL

**Affiliations:** 1 Department of Internal Medicine, Faculty of Medicine, Hacettepe University, Ankara, Turkey; 2 Department of Infectious Diseases and Clinical Microbiology, Faculty of Medicine, Hacettepe University, Ankara, Turkey

**Keywords:** *Bordetella pertussis*, polymerase chain reaction, surveillance

## Abstract

**Background/aim:**

*Bordetella pertussis* infection remains an important health problem in adults due to the increasing prevalence in recent years. Pertussis in adults can be easily overlooked or misdiagnosed. We aimed to determine the prevalence of pertussis in adult patients with acute cough and the clinical features of the pertussis cases.

**Materials and methods:**

Internal Medicine and Pulmonology inpatient wards and outpatient clinics were screened between March 2017 and June 2018. Patients with cough duration between 1 week and 1 month were enrolled. Those who were on antibiotics for more than 5 days were excluded. A total of 115 patients were enrolled. Nasopharyngeal swabs were taken and real-time polymerase chain reaction analyses were done.

**Results:**

According to the pertussis case definition, 47.8% of the patients were diagnosed with probable pertussis. We found the prevalence of pertussis as 3.5% in our cohort. All of the patients with pertussis had a history of paroxysmal cough with a mean duration of 20 days.

**Conclusions:**

These results show that pertussis continues to be a health problem for adults and may present with acute cough. The growing number of adult pertussis cases suggest that vaccination of children is inadequate to prevent pertussis and the concept of ‘lifelong vaccination’ should be strengthened.

## 1. Introduction

Whooping cough, which we know for more than a century, is a highly contagious and life-threatening respiratory system disease and has an impact especially on newborns, pregnant women, and elderly. The connection between pertussis and the causative bacteria was established in 1900 and the whole-cell pertussis vaccine was first introduced in the 1930s [1]. Since then, there have been great advances in controlling the disease spread, however, pertussis continues to be a problem for vulnerable individuals.

**Figure 1:**
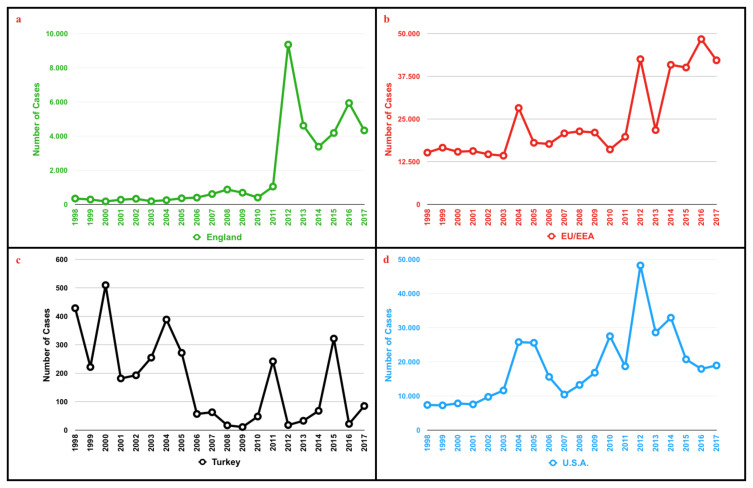
Number of reported pertussis cases over the past 2 decades, a: England; data derived from laboratory confirmed cases of pertussis in England from 1994 to 2017. Pertussis annual report, b: The European Union and European Economic Area (EU/EEA) countries; data derived from European Centre for Disease Prevention and Control (ECDC), Surveillance ATLAS of Infectious Diseases, surveillance and disease data, c: Turkey; data derived from WHO vaccine-preventable diseases: monitoring system. 2020 global summary incidence time series for Turkey, d: USA; data derived from CDC Surveillance and Reporting; pertussis cases by year.

**Figure 2:**
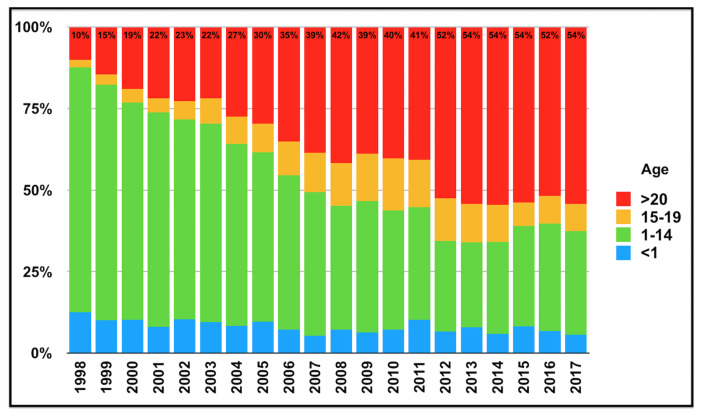
Age distribution of reported pertussis cases over the past 2 decades, The European Union and European Economic Area (EU/EEA) countries; data derived from European Centre for Disease Prevention and Control (ECDC), Surveillance ATLAS of Infectious Diseases, surveillance and disease data.

Although the incidence of pertussis decreased dramatically with childhood vaccination programmes, in recent years there has been an increase in the incidence worldwide. It is thought that waning immunity, shift from a whole-cell vaccine to an acellular vaccine, active surveillance, better diagnostic tests and increased awareness might be the possible causes of this increase [2–4]. According to the data of the Centers for Disease Control and Prevention (CDC) in Washington, the highest number of case reports were announced in 2012, 70 years after 1942 [3]. The number of cases reported in England in the last decade has also increased^1^. The number of reported pertussis cases over the past two decades for England^1^, The European Union and European Economic Area (EU/EEA) countries^2^, Turkey^3^ and United States^4^ are shown respectively in Figure 1a, 1b, 1c and 1d. In the past, infants used to constitute the majority of cases. However, in recent years there is a significant increase in the prevalence of pertussis among adolescents and adults in many countries. Age distribution of reported pertussis cases in EU/EEA countries^2^ is shown in Figure 2.

Polymerase chain reaction (PCR) is the most sensitive method for rapid detection of *Bordetella pertussis*. However, as is the case with the gold standard method, culture, its sensitivity decreases over time as the duration of cough increases. PCR is a helpful diagnostic test until the 4^th^ week of cough. However, the use of PCR for diagnosis is not recommended after the fifth day of the antibiotic use, as antibiotic use may cause false-negative results [5]. After the inclusion of PCR test results to pertussis case definition, PCR use became more widespread, and in 2012 more than 84% of cases in the United States were diagnosed with PCR [6].

It is recommended that milder cases with few symptoms and short-term cough should be included in studies identifying *B. pertussis* infections, since the disease may demonstrate an atypical course in some individuals. Active surveillance studies suggest that pertussis frequency is much higher than that is reported. Many pertussis cases remain undiagnosed and cannot be reported due to atypical disease course and limitations in laboratory tests [7, 8]. This might well be the case in Turkey. Studies conducted in variable patient groups in different cities of Turkey showed that the prevalence of pertussis-positive cases can be as high as 26% [9,10].

The primary aim of this study was to determine the prevalence of pertussis in adult patients with acute cough. The second aim was to determine the clinical features, course, and outcome of the identified pertussis cases.

## 2. Materials and methods

The patients who were admitted to Internal Medicine inpatient wards of a University Hospital and those seen at the Internal Medicine and Pulmonology outpatient clinics of the same hospital between March 2017 and June 2018 were screened. Patients older than 18 years of age and with cough duration between 1 week and 1 month were enrolled in the study. Patients who were on antibiotics for more than 5 days, patients with cough duration less than 1 week and more than 1 month, and patients who did not consent were excluded. Nasopharyngeal swabs were taken from the patients who were eligible and who consented.

The most widely used case definition as accepted by the Council of State and Territorial Epidemiologists and used to report to the Centers for Disease Control and Prevention, which is also the reference in this study, is summarized in Table 1^5^.

Sterile single pack, thin tip, flexible, plastic swabs (FloqSwabsTM Flocked swab 503CS01, Copan Diagnostics Inc., USA) were used to collect nasopharyngeal specimens from patients. DNA extraction was performed with BD MAXTM ExKTM DNA-1 extraction kit from nasopharyngeal swab samples.

Multi-targeted BioGX Sample-ReadyTM open system reaction kits were used when performing Real Time-Polymerase Chain Reaction (RT-PCR), which can detect *Bordetella pertussis* (IS481), *Bordetella parapertussis* (pIS1001), *Bordetella holmesii* (hIS1001), pertussis toxin gene (ptxS1), the sample extraction control and a *Drosophila* (sample processing control, SPC) DNA as an internal amplification control. RT-PCR was performed with the automated system of the BD MaxTM System (Becton Dickinson, USA) and results were evaluated as recommended by the producer.

## 3. Results

There were 115 patients who met inclusion criteria and were enrolled in the study. The mean age of the patients was 46.4 years and 72 (62.6%) of the patients were female (Table 2). More than half of the patients had at least one chronic condition, the most prevalent one being hypertension. Most of the patients were recruited from the outpatient clinics. The duration of cough was lower than 21 days in 83 (72.2 %) of the patients (Table 2). According to CDC pertussis case definitions, 47.8% of the patients were diagnosed with probable pertussis. Paroxysms of cough were present in 73% of the enrolled patients.

Four patients were diagnosed to have pertussis with regards to *B. pertussis* PCR results, hence the prevalence of pertussis in this adult patient population with acute cough was 3.5%. All of the patients described paroxysms of cough and one patient described posttussive vomiting (Table 3). The fourth patient with pertussis, who was a 66-year-old woman with gastric cancer, was hospitalized for gastrointestinal bleeding and died on the 30^th^ day of her admission.

## 4. Discussion

Pertussis is a disease that may affect all age groups, although it is perceived as a childhood disease. Adults can be affected by atypical and mild forms of the disease, and they also act as an important source of infection for infants. Although, pertussis is well-known as an aetiological factor in adults with prolonged cough, we aimed to determine its prevalence in adult patients who present with acute cough and found a prevalence of 3.5%. Previous data have shown that the actual prevalence of pertussis might be quite high (Figure 1). Therefore, surveillance studies become more important in terms of demonstrating the burden of disease, determining the epidemiological trends and using resources more effectively. In this respect, our study showed that pertussis is one of the diseases that should not be neglected in the adult age group.

The prevalence of pertussis in different studies can be quite variable due to the selection criteria of the patients and the differences in the laboratory methods. The mean prevalence of pertussis was found to be 3% (0% to 6.2%) in adults with cough duration between 2 to 4 weeks in a European multicentre study [11]. In a study evaluating 214 adolescent and adult patients with cough duration of more than 2 weeks, pertussis PCR was found to be positive in 15 (7%) subjects [12]. Another study evaluating pertussis seroprevalence in adults, 52 (9.7%) of 538 patients with cough duration for more than 2 weeks suggested that their *B. pertussis* antibody levels were compatible with acute or recent infection [13].

Many health authorities believe that reported cases do not reflect actual numbers^6^ [14]. It has been postulated that reported pertussis cases are 40 to 160 times less than the actual disease incidence, and asymptomatic infections are 4 to 22 times more common than symptomatic infections [15]. The reported prevalence of pertussis was variable among studies in different age groups with different clinical presentations in our country, higher than 20% in many of them [9,10]. As Shown in Figure 1 and Figure 2, not only has there been an increase in the number of reported cases over the last 2 decades, but also there has been a change in the age distribution of the reported cases. In the early 2000s, only 5%–10% of pertussis cases reported in the United Kingdom were older than 24 years of age, but in recent years this rate is over 50%^7^. A similar change is also seen in Denmark, where the proportion of the over 20-year-olds in the distribution of laboratory-verified cases increased from 14% to 43% from 1995 to 2013 [16].

Studies evaluating transmission pathways of pertussis in children indicate that adolescents and adults are reservoirs for pertussis [17]. The sources of pertussis infection were adults, mainly the parents and in-household contacts, in 70%–80% of paediatric pertussis cases [17]. It is evident that adults are the major source of infection who transmit pertussis to babies, who have the highest morbidity and mortality. Neither having pertussis infection nor having pertussis vaccine does provide life-long protection. Immunity gained in both ways decreases with time [15]. Although the three doses of primary pertussis vaccination coverage among children under 1 year of age have been 96% and over in Turkey, seroepidemiological data from our country demonstrated that adults constitute a potential reservoir for pertussis^8^. It was shown that 90% of adults that presented to the outpatient clinic did not have protective antibody levels for pertussis [18]. Thus, a lifelong vaccination strategy for pertussis should be established. Many guidelines now recommend to have a pertussis booster dose in adulthood in a way that one of the tetanus-diphtheria boosters to be replaced by tetanus-diphtheria-acellular pertussis booster^9,10,11^.

In our study, almost half of patients presenting with acute cough were diagnosed as probable pertussis cases with clinical criteria. Vittucci and colleagues found that the sensitivity of the clinical definition in diagnosing pertussis cases was 30.2% in infants [19], thus, it is not possible to exclude the diagnosis of pertussis by clinical features. On the other hand, pertussis is a disease that can easily be overlooked as well [19,20]. We found that the mean duration of paroxysmal cough within pertussis positive cases was 20 days. It is known that there may be delays in the diagnosis because of not considering pertussis as a priority in differential diagnosis even if the patients are admitted early [19]. Heil and colleagues found that the median time from the onset of the disease until the diagnostic test request was 28 days (21–47 days) [21]. Early detection and proper treatment of pertussis can reduce the transmission of the infection [21].

One of the limitations of our study was that the patients were not screened by any laboratory method other than PCR. Although patients with cough duration of more than 4 weeks were excluded due to decreasing sensitivity of PCR, it still might have yielded false-negative or false-positive results. In addition, we did not make analyses for other viral and bacterial aetiologies, hence we could not point out the proportion of pertussis cases among other microbial aetiologies. On the other hand, the strengths of the study were its surveillance methodology and the utilization of PCR. All patients presenting with cough disease were prospectively screened and those who fulfilled enrolment criteria were screened by PCR, which is a fast and highly sensitive diagnostic method.

In conclusion, we demonstrated that in a cohort of patients presenting with acute cough, half of the patients were diagnosed as probable pertussis cases with clinical criteria and the prevalence of pertussis was found to be 3.5%. These results indicate that pertussis can be an aetiological factor in patients presenting with acute cough and real-time laboratory tests are needed to verify probable cases as confirmed cases. Finally, training programmes to increase pertussis awareness and reinforce the concept of ‘lifelong vaccination’ will help to decrease the burden of pertussis among adults and its transmission to vulnerable infants.

## Figures and Tables

**Table 1:** Pertussis case classification.

Criteria		Confirmed cases			Probable cases			
Criteria group	Definition	1	2	3	1	2*	3*	4
**Clinical criteria**	Acute cough illness (any duration)	+				+	+	
	Cough ≥2 weeks duration		+	+	+			+
	Inspiratory whoop		o	o	o	o	o	o
	Paroxysms of coughing		o	o	o	o	o	o
	Posttussive vomiting		o	o	o	o	o	o
	Apnoea (with or without cyanosis), (For infants <1 year of age only)		o	o	o	o	o	o
**Laboratory criteria**	Isolation of *B. pertussis* from a clinical specimen	+						
	Positive PCR for *B. pertussis*		+			+		
**Epidemiological linkage**	Contact with a laboratory-confirmed case (classified as “confirmed”)			+			o	
	Contact with a laboratory-confirmed infant case (classified as “probable”)						o	+

* For infants <1 year of age only

**Notes:** + = All “+” criteria in the same column are necessary to classify a case. o = At least one of these “o” (Optional) criteria in each category (e.g., clinical criteria and laboratory criteria) in the same column—in conjunction with all “+” criteria in the same column—is required to classify a case.

Data derived from Council of State and Territorial Epidemiologists Position Statement^1^

**Table 2:** Demographic characteristics and clinical features of patients with acute cough disease (n = 115).

Characteristics		n (%)
**Age**		46.4 (18.6) *
**Female gender**		72 (62.6)
**Medical history**	No chronic illness	49 (42.6)
	Hypertension	31 (27.0)
	Diabetes mellitus	17 (14.8)
	Chronic lung disease	28 (24.3)
	Coronary artery disease	11 (9.6)
	Malignancy	11 (9.6)
	Other chronic conditions**	24 (20.9)
**Smoking**		34 (29.6)
**Type of admission**	Outpatient	99 (86.1)
	Inpatient	16 (13.9)
**Duration of cough**	7–14 day	38 (33.0)
	14–21 day	45 (39.1)
	21–28 day	32 (27.8)
**Characteristics of cough**	History of contact with a coughing person before illness	21 (18.3)
	Paroxysm	84 (73.0)
	Vomiting after coughing	10 (8.7)
	Dyspnoea	62 (53.9)
	Chest pain	39 (33.9)
	Fever	28 (24.3)
	Headache	47 (40.9)
	Disturbance of sleep	55 (47.8)
	Disturbance of daily activities	40 (34.8)

Notes: Values are given in numbers and percent n (%) if otherwise specified, one patient might have more than one chronic condition.

* mean (standard deviation)

** chronic kidney disease, chronic liver disease, heart failure, heart valve disorders, rheumatological diseases, hypothyroidism, cerebrovascular disease, cardiac arrhythmia, Parkinson disease, and thrombophilia

**Table 3:** Characteristics of patients with a positive *Bordetella* PCR.

Patient	Age	Sex	Duration of cough (day)	Paroxysms	Posttussive vomiting	Disturbance of sleep and daily activities	Medical history	Inpatient/ outpatient
**1** *	34	M	18	Yes	No	Yes	No chronic illness	Outpatient
**2**	69	F	28	Yes	No	Yes	HT	Outpatient
**3**	34	F	20	Yes	No	Yes	No chronic illness	Outpatient
**4** **	66	F	15	Yes	Yes	Yes	Gastric cancer	Inpatient

* He was an officer in a high school.

** Hospitalized in the intensive care unit because of gastrointestinal bleeding and died in the hospital.

F: female, M: male, PCR: polymerase chain reaction HT: hypertension

## Footnotes

1 Public Health England (2018). Laboratory confirmed cases of pertussis in England from 1994 to 2017. Pertussis annual report: supplementary data tables 2017. [online]. Website https://www.gov.uk/government/publications/whooping-cough-pertussis-statistics [Accessed: 14 March 2020].

2 European Centre for Disease Prevention and Control (2020). Surveillance and Disease Data: Surveillance ATLAS of Infectious Diseases. [online]. Website https://atlas.ecdc.europa.eu/public/index.aspx [Accessed: 14 March 2020].

3 World Health Organization (2020). WHO vaccine-preventable diseases: monitoring system. 2020 global summary, Incidence time series for Turkey. [online]. Website http://apps.who.int/immunization_monitoring/globalsummary/incidences?c=TUR [Accessed: 15 March 2020].

4 U.S. Department of Health and Human Services Centers for Disease Control and Prevention (2020). Pertussis Surveillance and Reporting: Pertussis Cases by Year. [online]. Website https://www.cdc.gov/pertussis/surv-reporting/cases-by-year.html [Accessed: 14 March 2020].

5 Council of State and Territorial Epidemiologists Position Statement (2013). Revision of the pertussis surveillance case definition to more accurately capture the burden of disease among infants <1 year of age. [online]. Website https://cdn.ymaws.com/www.cste.org/resource/resmgr/PS/13-ID-15.pdf [Accessed: 05 January 2022].

6 World Health Organization (2001). World Health Organization Department of Vaccines and Biologicals, Pertussis Surveillance: A Global Meeting Geneva. [online]. Website https://apps.who.int/iris/bitstream/handle/10665/66828/WHO-VB-01.19-eng.pdf?sequence=1&isAllowed=y [Accessed: 07 July 2019].

7 Public Health England (2018). Laboratory confirmed cases of pertussis in England from 1994 to 2017. Pertussis annual report: supplementary data tables 2017. [online]. Website https://www.gov.uk/government/publications/whooping-cough-pertussis-statistics [Accessed: 14 March 2020].

8 World Health Organization (2020). WHO vaccine-preventable diseases: monitoring system. 2020 global summary, Coverage time series for Turkey, [online]. Website https://apps.who.int/immunization_monitoring/globalsummary/coverages?c=TUR [Accessed: 15 March 2021].

9 Advisory Committee on Immunization Practices (2021). Recommended Adult Immunization Schedule for ages 19 years or older, United States. [online]. Website https://www.cdc.gov/vaccines/schedules/hcp/imz/adult.html?CDC_AA_refVal=https%3A%2F%2Fwww.cdc.gov%2Fvaccines%2Fschedules%2Fhcp%2Fadult.html [Accessed: 15 March 2021].

10 Türkiye Enfeksiyon Hastalıkları ve Klinik Mikrobiyoloji Uzmanlık Derneği, Erişkin Bağışıklama Rehberi (2019). [online]. Website https://www.ekmud.org.tr/haber/264-turkiye-ekmud-eriskin-bagisiklama-rehberi. [Accessed: 15 March 2021].

11 Global Strategy for the Diagnosis, Management, and Prevention of Chronic Obstructive Pulmonary Disease 2021 Report. [online]. Website https://goldcopd.org/wp-content/uploads/2020/11/GOLD-REPORT-2021-v1.1-25Nov20_WMV.pdf [Accessed: 15 March 2021].

1 Council of State and Territorial Epidemiologists Position Statement (2013). Revision of the pertussis surveillance case definition to more accurately capture the burden of disease among infants <1 year of age. [online]. Website https://cdn.ymaws.com/www.cste.org/resource/resmgr/PS/13-ID-15.pdf [Accessed: 05 January 2022].
